# Tibia stress injury and the imaging appearance of stress fracture in juvenile dermatomyositis: six patients’ experiences

**DOI:** 10.1186/s12969-021-00501-9

**Published:** 2021-02-17

**Authors:** Tomo Nozawa, Audrey Bell-Peter, Andrea S. Doria, Jo-Anne Marcuz, Jennifer Stimec, Kristi Whitney, Brian M. Feldman

**Affiliations:** 1 Division of Rheumatology, Department of Pediatrics, The Hospital for Sick Children, 555 University Ave, M5G 1X8 Toronto, ON Canada; 2 Department of Pediatrics, Yokohama City University Graduate School of Medicine, 3-9 Fukuura, Kanazawa-ku, Yokohama, Japan; 3 Department of Diagnostic Imaging, The Hospital for Sick Children, University of Toronto, 555 University Ave, M5G 1X8 Toronto, ON Canada; 4 Institute of Health Policy, Management and Evaluation, Dalla Lana School of Public Health, University of Toronto, 155 College Street, M5T 3M6 Toronto, Toronto, ON Canada

**Keywords:** Complication, Juvenile dermatomyositis, Stress fracture, Stress reaction, Tibia, Treatment

## Abstract

**Background:**

Tibial stress injuries are frequent injuries of the lower extremity and the most common causes of exercise-induced leg pain among athletes and military recruits. They sometimes occur in patients with pathological conditions of bone metabolism such as osteoporosis or rheumatoid arthritis, but there are previously no cases reported in juvenile dermatomyositis (JDM). Here we report 6 JDM patients who presented with shin pain, and the imaging appearance of tibial stress fractures or stress reactions.

**Case presentation:**

All 6 patients with JDM presented with shin pain or tenderness in the anterior tibia without any evidence of excessive exercise or traumatic episode. They were diagnosed with tibial stress injuries based on a combination of radiographs, three-phase bone scans, and magnetic resonance imaging (MRI), and 5 out of 6 patients had been treated with prednisone and/or methotrexate at onset of tibial stress injuries. In one patient, we could not find any abnormalities in his radiograph, but the subsequent MRI showed tibial stress reaction. In all 6 patients, the tibial stress injuries improved with only rest and/or analgesics.

**Conclusion:**

We experienced 6 children with JDM who presented with shin pain, and who were diagnosed with tibial stress fractures or stress reactions. Their underlying disease and weakness, treatment with glucocorticoid and methotrexate, or inactivity may have resulted in these tibial injuries, and made these patients more predisposed than other children. In addition to preventing JDM patients from getting osteoporosis, we need to consider stress reactions when children with JDM complain of sudden shin pain.

## Background

Juvenile dermatomyositis (JDM) is a rare chronic inflammatory disease of childhood, characterized by skin involvement and proximal muscle weakness.

When JDM patients complain of pain in the lower extremities, pediatric rheumatologists may initially consider this to be an exacerbation of myositis. However, when leg pain – especially shin pain – appears, there are some differential diagnoses in addition to myositis. For example, tibial stress fractures and stress reactions – also known as “shin splints” – should be considered as one of the differential diagnoses. Generally, stress fractures are common in athletes and military recruits, and the most common location of stress fracture in the general population is the tibia [1]. This is also found to be the most common location among older children, in whom the most prevalent site is the proximal tibia [2–4]. Besides athletes, some reports describe stress fractures occurring in patients with pathological conditions of bone metabolism such as osteoporosis, or in rheumatoid arthritis [5]. However, there are no cases reported in the literature of this occurring in JDM. Here we report 6 JDM patients who presented with shin pain, and the imaging appearance of tibial stress fractures or stress reactions.

## Case presentation

Table 1 shows the clinical features of 6 JDM patients who were diagnosed with tibial stress fractures or stress reactions. All imaging of those patients was reviewed by 2 radiologists, based on the Fredericson classification system [6]. Stress fracture was diagnosed by sclerosis, periosteal reaction/elevation, cortical thickening/or a fracture line at the site of pain. Stress reaction was defined as findings of bone marrow edema at a symptomatic site with no definite fracture on radiography or MRI [7].

**Table 1:** Main clinical features of the 6 patients

	Case 1	Case 2	Case 3	Case 4	Case 5	Case 6
Sex	Female	Female	Male	Male	Male	Female
Age at stress reaction/stress fracture (years)	10	5	9	4	5	11
Months since diagnosis of JDM (months)	3	Prior to JDM diagnosis	7	18	9	20
Symptoms	Right lower leg pain	Tenderness over the right shin	Bilateral knee pain	Right shin pain	Bilateral shin pain	Right shin pain
Stress reaction or stress fracture	Stress fracture	Stress fracture	Stress reaction	Stress reaction	Stress reaction	Stress fracture
Location of stress reaction/stress fracture	Middle and lower third of right tibia, right proximal fibula	Bilateral proximal tibiae	Middle third of bilateral tibiae	Middle diaphysis of the right tibia	Bilateral anterior cortical tibiae	Middle diaphysis of the right tibia
JDM therapy at stress reaction/stress fracture	Prednisone 1.2 mg/kg/day	No treatment	Prednisone 0.6 mg/kg/day, MTX	MTX	MTX	MTX
Interventions for stress injury/stress fracture	Rest and analgesics	Rest	Rest	Rest	Rest	Rest
Physical function before tibial lesion	Limitation of extracurricular activities only	Limitation of instrumental daily activities	No limited activities	No limited activities	Limitation of extracurricular activities only	No limited activities
Muscle weakness in lower extremities at time of tibial lesion	No	Yes	No	No	Yes	No
Modified disease activity score at stress reaction/stress fracture (0–11)	2	Not evaluated	1	1	3	3
Age matched BMD	Normal range	Normal range	Normal range	Not evaluated	Normal range	Normal range
Regional osteoporosis or osteopenia	No	No	No	Osteopenia	No	No
Serum 25-hydroxyvitamin D prior to stress reaction/stress fracture, nmol/L	65	Not measured	19	113	100	45

Modified disease activity score includes clinical parameters for three dermatological (0–4 points) and three musculoskeletal criteria (0–7 points) [24]. *BMD* Bone mineral density, *JDM* Juvenile dermatomyositis, *MTX* Methotrexate

### Case 1

This girl was diagnosed at the age of 9 with JDM based on proximal muscle weakness, arthritis, and the appearance of a heliotrope rash and Gottron papules. After making a diagnosis, she was started on prednisone at a dose of 2 mg/kg/day. She showed significant improvement in her symptoms, except for persistent skin manifestations. Her prednisone was gradually tapered. Three months later, right lower shin pain suddenly appeared without any evidence of excessive exercise or traumatic episode. She presented for evaluation 3 days later at which time she had only right lower shin pain and no other abnormal symptoms or signs. A radiograph performed at another hospital revealed a horizontal line at the junction of the middle and lower thirds of the right tibia, consistent with a stress fracture. One week later, on radiographic re-examination and three-phase bone scan, an undisplaced fracture at the right proximal fibula was revealed. Her right lower shin pain improved with conservative management of short-term rest and analgesics by 2 months. Bone mineral density (BMD), by dual energy X-ray absorptiometry (DEXA) of both the regional bone and the lumbar spine (83 % of age-matched BMD) was in the normal range.

### Case 2

This 5 year-old girl had some tenderness, lasting for 2 weeks, in the anterior right tibia 4 months before a diagnosis of JDM was made, based on rash and muscle weakness with an abnormal muscle biopsy. She had never previously received any treatment with glucocorticoids or disease-modifying anti-rheumatic drugs. At the same time as she developed tenderness in her right tibia, she also presented with fatigue, anorexia, and diffuse joint and muscle pains. At the time of evaluation for JDM, a radiograph and three-phase bone scan were performed looking for the cause of her tibial tenderness. The radiograph of her right tibia revealed thickening of the anterior cortex of the mid tibia. Furthermore, the three-phase bone scan showed a focal linear increased uptake along the lateral cortex of the proximal tibia bilaterally. There was no evidence of any soft tissue uptake. The findings of the radiograph and three-phase bone scan were consistent with stress fractures of both tibiae. When she was transferred to our hospital six months later, the fractures had already healed without additional treatment. A BMD scan looking at regional bone and both femoral necks was normal at the time of transferring her care to our hospital.

### Case 3

**Fig. 1:**
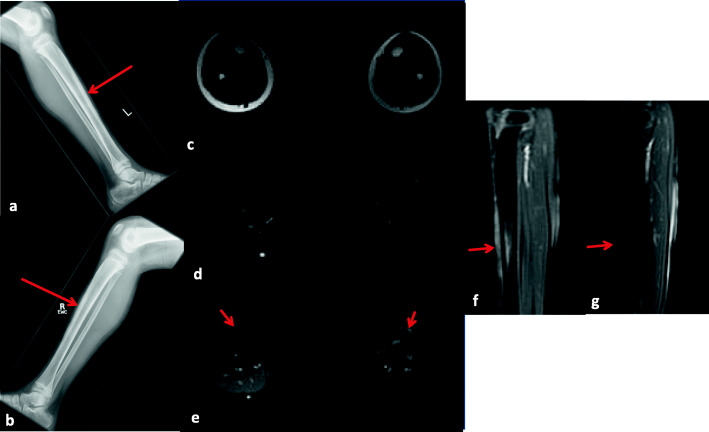
Case 3 Lateral radiographs of the left (**a**) and right (**b**) lower extremities (**a**, **b**) show prominence of the anterior mid cortex of bilateral tibias without associated periosteal reaction or bone abnormalities (arrows) Axial T1 (**c**), fat saturated T2 (**d**) and post-gadolinium T1 (**e**) MR images, and sagittal post-gadolinium T1 (**f**, left; **g**, right) MR images with the lower extremities were obtained 2.5 months after the aforementioned x-rays. These images show bilateral anterior periosteal thickening, increased T2 signal intensity and enhancement noted within the bone marrow and anterior soft tissues of the mid third of both tibias (arrows), more prominent on the left than on the right

This boy was diagnosed with JDM on magnetic resonance imaging (MRI) at the age of 9 when he presented with polyarthralgia, vasculopathic rash and proximal muscle weakness. He was initially treated with prednisone at a dose of 1 mg/kg/day and methotrexate (MTX). With this treatment, his symptoms associated with JDM improved significantly. As a result, he was able to gradually taper his prednisone without JDM flare. Six months after diagnosis, severe bilateral shin pain lasting for 2 weeks appeared suddenly without muscle weakness and joint involvement. Although he was regularly playing basketball – due to increased physical functional capacity – before the pain episode, he had no recalled trauma. The physical examination revealed mild tenderness over both of his shins. Radiographs, an MRI and a three-phase bone scan were performed. The radiographs showed no evidence of fracture or periosteal reaction in the tibia or fibula, but the subsequent MRI showed bilateral periosteal thickening, and increased T2 signal intensity within the bone marrow and anterior soft tissues of the middle third of both tibias (Fig. 1). In addition, the three-phase bone scan revealed bilateral symmetric linear increased activity that was noted in the anterior tibial cortices on blood pool and (more intensely) on delayed images. These findings were consistent with a tibial stress reaction. His shin pain improved without analgesics over several months. A BMD of the regional bone was normal, and BMD in the lumbar spine and femur was normal (96 % and 92 % of age matched BMD values, respectively).

### Case 4

**Fig. 2:**
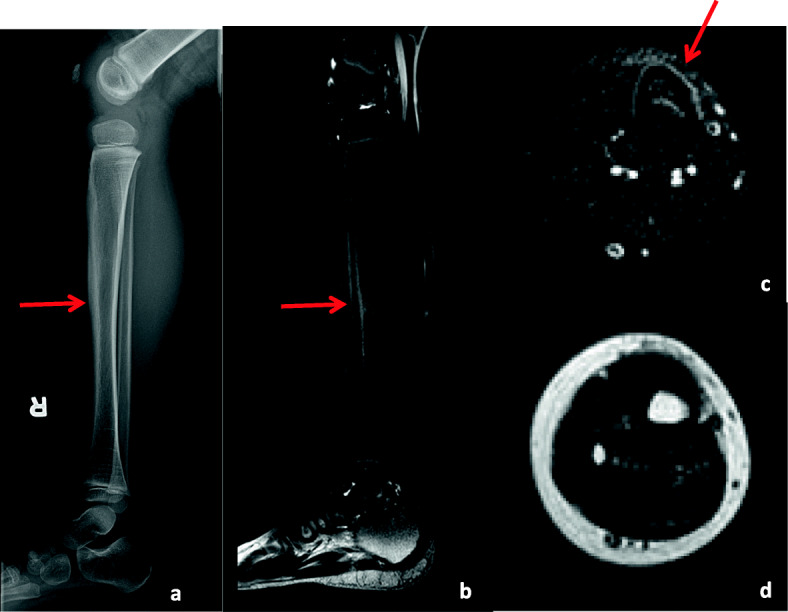
Case 4 Lateral radiograph of the right lower extremity (**a**) shows linear subperiosteal new bone formation along the mid tibial diaphysis The fat-saturated sagittal (**b**) and axial (**c**) T2 MR images of the right lower extremity obtained 4 months after the aforementioned x-rays show periosteal reaction along the mid right tibial diaphysis associated with increased signal intensity within the underlying cortex and bone marrow and at a lesser extent, anterior soft tissues (arrows). Low signal intensity is noted in the corresponding regions of the axial T1 (**d**) MR image. No discrete fracture line is noted

This boy was diagnosed with JDM at the age of 3 based on Gottron papules, polyarthritis, muscle weakness with elevated creatinine kinase, and MRI abnormalities indicating myositis. He was initially treated with prednisone at a dose of 2 mg/kg/day and MTX. With this treatment his symptoms improved, except for mild persistent skin manifestations; he was able to discontinue prednisone 10 months after starting treatment. Five months later, he developed right shin pain lasting for 2 weeks, that suddenly started without any recalled trauma. He had tenderness, swelling and warmth over the right tibia. Radiographs demonstrated linear subperiosteal new bone formation adjacent to the middle diaphysis with decreased bone density (Fig. 2). Further evaluation by MRI showed periosteal reaction with edematous changes in his right tibia without a discrete fracture line, and without worsening inflammation of the surrounding muscles (Fig. 2). His findings were consistent with a tibial stress reaction. His shin pain improved without analgesics after about 5 months. He did not have BMD evaluation by DEXA during his investigation in our institution.

### Case 5

This boy was diagnosed with JDM at the age of 4 based on Gottron papules, heliotrope rash, and proximal muscle weakness with MRI findings. He was initially treated with prednisone at a dose of 2 mg/kg/day and MTX. His JDM activity remained controlled during tapering of his prednisone and he was able to discontinue his prednisone 8 months after starting. One month later, he developed bilateral shin pain, lasting for 3 weeks, that was associated with restriction in his exercise capacity and interference with sleep. He recalled no traumatic episodes. The physical examination found point tenderness in the mid one-third of the left tibia. Radiographs and MRI showed thickening / periosteal reaction noted at the anterior cortex of the bilateral tibias, consistent with a tibial stress reaction. There was minimal bone marrow edema at the anterior part of the bones, but no associated myositis or obvious fracture. Five months later, his shin pain resolved with conservative measures and rest. A BMD of the regional bone and the lumbar spine (92 % of age matched BMD values) was normal.

### Case 6

**Fig. 3:**
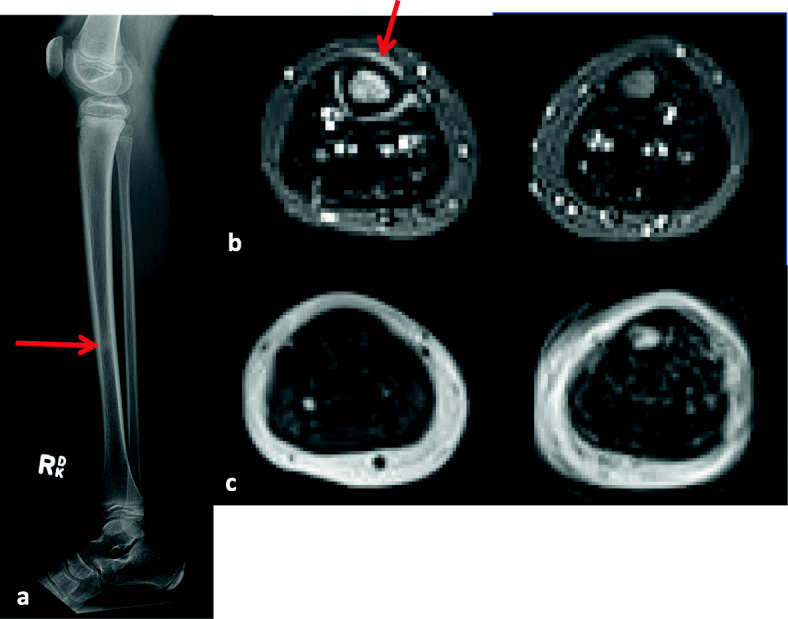
Case 6 (Initial observation) Lateral radiograph of the right lower extremity (**a**) shows a focal area of sclerosis in the mid tibial diaphysis with no associated periosteal or cortical thickening (arrow) Axial inversion recovery MR images of the thighs (**b**) obtained 2 weeks after the aforementioned x-rays show a focus of increased bone marrow signal in the mid right tibial diaphysis associated with a hyperintense periosteal halo on fluid sensitive images (arrow) which presents with low signal intensity on corresponding axial T1 images (**c**)

**Fig. 4:**
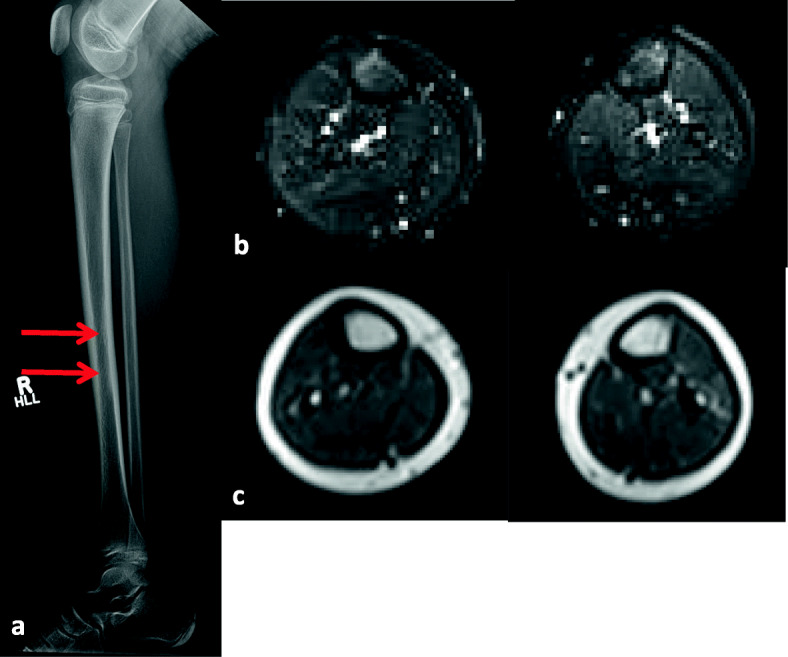
Case 6 (Follow-up) Follow-up lateral radiograph of the right lower extremity (**a**) obtained 3 months after the initial x-rays shows persistence of a focal area of sclerosis in the mid tibial diaphysis (arrows), increased in extent compared to previous. Subsequent axial inversion recovery (**b**) and T1 (**c**) MR images of the thighs obtained almost 1 year after the initial x-rays show interval resolution of the previously noted focus of increased bone marrow signal in the mid right tibial diaphysis

This girl was diagnosed with JDM at the age of 9 based on Gottron papules, heliotrope rash, and proximal muscle weakness, with elevated creatinine kinase and typical MRI findings. She was initially treated with prednisone at a dose of 2 mg/kg/day and MTX. She responded well and was able to discontinue prednisone 1 year after starting. Seven months later, she developed right shin pain, lasting for about 5 weeks, that appeared without any trauma. Due to increasing pain, radiographs were performed, followed by a subsequent MRI scan. Her radiographs were suggestive of a previous stress injury in the mid tibial diaphysis. The MRI examination showed altered marrow signal within the right tibia, demonstrating T1 hypointensity and T2 hyperintensity, consistent with a stress fracture (Fig. 3). The follow-up lateral radiograph which was obtained 3 months after the initial X-ray showed persistence of a focal area of sclerosis, increased in extent compared to previous (Fig. 4). However, the shin pain gradually improved within 6 months, and a follow-up MRI examination which was performed almost 1 year after the initial X-ray showed improvement of the altered marrow signal (Fig. 4). BMD of the regional bone and lumbar spine (84 % of age matched BMD values) was normal.

## Discussion and conclusions

Stress fractures in prepubescent children who don’t vigorously participate in sport activities appear to be very uncommon. In the pediatric age group, there are only a few case reports and small series, mostly of child athletes. In a large population-based study, it was reported that 3.9 % of adolescent girls, aged 9 to 15 years, developed a stress fracture during a 7-year period, and that these stress fractures were related to participating in running, basketball, cheerleading, and gymnastics [8].

As our case series is retrospective, it is unclear whether children with JDM are more susceptible to tibial stress fractures and tibial stress reactions compared to healthy children. However, tibial stress fractures and tibial stress reactions developed in 6 of 189 patients (3.2 %) who were followed in our inception cohort and met the 2017 EULAR/ACR classification criteria [9]; while this frequency is similar to that reported by Field et al. [8], our patients were mostly not athletic at the time of injury, being in the recovery phase of myositis.

It may be that JDM poses additional risks for tibial stress fractures / stress reactions. First, stress fractures appear to occur because of repetitive muscular forces or stresses applied to a bone. Previous papers have reported muscle imbalance and inflexibility, especially tightness of the triceps surae (soleus, gastrocnemius, and plantaris muscles), as being commonly associated with tibial stress reactions [10]. For example, athletes with muscle weakness of the triceps surae tend to develop muscle fatigue, leading to altered running mechanics, and strain on the tibia. Also, weakness of the core muscles is reported to be an important risk factor for lower extremity injuries. Hip and pelvis muscle strength are an important link in maintaining control and proper mechanics between the core and lower extremity. Children with JDM who have muscle weakness or insufficient muscle endurance may, therefore, be predisposed to develop tibial stress reactions. Second, glucocorticoid and/or MTX treatment has been suggested as a possible cause for stress fractures and stress reactions in the tibia [11–15]. Both medications are commonly known to induce reduced bone density. MTX can cause osteopathy in two ways; MTX increases urinary and fecal excretion of calcium and enhances osteoclastic bone resorption [16]. A study of MTX given to rats over the short-term demonstrated toxic effects on osteoblasts, with a 60 % reduction on the rate of bone formation, resulting in decreased osteoid volume and thickness [17]. In previous reports that have described tibial stress reactions in patients with rheumatoid arthritis and psoriasis, those patients were treated with methotrexate and/or glucocorticoid [13, 18]. Radiographs of the lesions in those patients demonstrated osteopenia, or osteoporosis, with the stress reactions. Only one of our patients (Case 4) had demonstrable regional osteopenia. Although withdrawal of MTX is the suggested choice when dealing with MTX osteopathy, our two JDM patients’ complications healed without stopping MTX. Except for Case 2 who had stress fractures before a diagnosis of JDM, 5 patients had received calcium and vitamin D supplementation to avoid osteoporosis since their diagnosis of JDM. Therefore, as shown in Table 1, the levels of serum 25-hydroxyvitamin D prior to stress reaction/stress fracture in 3 of 5 patients were within normal range (> 50 nmol/L). According to international guidelines, for glucocorticoid-treated children, calcium and vitamin D intake is recommended to avoid osteoporosis [19]. As a preventive strategy for stress fracture, a routine DEXA is reported to be very important in addition to calcium and vitamin D supplementation [20].

It would seem to be important to properly diagnose tibial stress fractures and stress reactions, both to rule out more sinister causes, but also because these stress fractures are sometimes a cause of disordered bone growth [10, 21]. The diagnosis requires a high level of clinical suspicion, based on focal tenderness of the tibia, and confirmation is needed with imaging [22]. Radiographs are sometimes normal until 3 weeks to 3 months after the onset of pain. We could not make the diagnosis of a stress fracture in one patient of our cohort (Case 3) when considering the initial radiograph solely. If the initial radiographs are normal but there is persistent clinical concern, MRI or three-phase bone scan should be considered for further diagnosis.

The most important management of tibial stress fracture and stress reaction is to reduce impact loading until the pain resolves. Even after these injuries resolve, though, care must be taken to detect the appearance of new lower extremity symptoms because of a high reported recurrence rate [23].

In conclusion, we have reported the cases of 6 children with JDM who presented with shin pain, and who were diagnosed with tibial stress fractures or stress reactions. Their underlying disease and weakness, treatment with glucocorticoid and MTX, or inactivity may have resulted in these tibial injuries, and made these patients more predisposed than other children. It would appear to be reasonable to follow international guidelines to avoid osteoporosis, in part to avoid these injuries [19]. Furthermore, we need to consider stress reactions when children with JDM complain of sudden shin pain.

## Data Availability

All data generated or analyzed during this study are included in this published article.

## Abbreviations

BMD: Bone mineral density
DEXA: Dual energy X-ray absorptiometry
JDM: Juvenile dermatomyositis
MRI: Magnetic resonance imaging
MTX: Methotrexate
